# Clinical and prognostic profile of Her2neu positive (non-luminal) intrinsic breast cancer subtype: comparison with Her2neu positive luminal breast cancers

**DOI:** 10.1186/s13104-018-3677-y

**Published:** 2018-08-13

**Authors:** Atif Ali Hashmi, Raeesa Mahboob, Saadia Mehmood Khan, Muhammad Irfan, Mariam Nisar, Narisa Iftikhar, Maham Siddiqui, Naveen Faridi, Amir Khan, Muhammad Muzzammil Edhi

**Affiliations:** 1 Department of Histopathology, Liaquat National Hospital and Medical College, Karachi, Pakistan; 2 Department of Medicine, Kandahar University, Kandahar, Afghanistan; 3 Department of Surgery, Brown University, Providence, RI USA

**Keywords:** Her2neu, Intrinsic breast cancer, ER, PR, Her2neu

## Abstract

**Objective:**

Her2neu receptor is proto-oncogene which can be over-expressed in both luminal and non-luminal breast cancers. In the present study, we aimed to compare the prognostic and predictive factors like tumor grade, T-stage, N-stage and ki67 index in Her2neu intrinsic breast cancer subtype with Her2neu over-expressed luminal breast cancers.

**Results:**

801 (41%) cases were Her2neu positive; out of which, 418 cases (52.2%) showed ER positivity and thus were classified as Her2neu positive luminal cancers whereas 383 cases (47.8%) were ER and PR negative and therefore were labeled as intrinsic Her2neu subtype (non-luminal). Her2neu positive (non-luminal) cancers were significantly associated with higher grades and Ki67 proliferative index compared to Her2neu positive luminal cancers. On the other no significant association was noted in T-stage and N-stage. We found a high frequency of her2neu positivity in our studied population of breast cancer. Moreover, association of her2neu positive (non-luminal) breast cancers with higher grade and ki67 index indicates a predictive value of ER/PR positivity in her2neu positive breast cancers. On the other hand, lack of association with respect to T and N stage, signifies no prognostic benefit of ER/PR in her2neu positive breast cancers.

## Introduction

Her2neu receptor is a proto-oncogene located on long arm of chromosome 17 with intracellular tyrosine kinase activity [1, 2]. It belongs to epidermal growth factor receptor family and is expressed at low levels in many normal tissues of the body where it drives epithelial cell growth [3, 4]. Over-expression of Her2neu occurs in 18–20% of breast cancers [5–7]. Gene expression profiling defines breast cancer subtypes on the basis of expression patterns of different genes. High expression of Her2neu gene, in the absence of estrogen receptor (ER) and progesterone receptor (PR) related genes are categorized as Her2neu intrinsic breast cancer subtype. On the other hand co-expression of Her2neu and ER related gene (although low) may be seen in luminal B subtype of breast carcinoma. Luminal B cancers with Her2neu positivity were found to be significantly associated with high tumor grade and higher frequency of nodal metastasis [8]. St. Gallen International Expert Consensus on the Primary Therapy of Early Breast Cancer 2013 proposed that intrinsic molecular subtypes of breast cancer can be defined without molecular diagnostics with the help of immunohistochemistry (IHC) for ER, PR, HER2neu and ki67 [9]. High expression of Her2neu is known to associated with poor prognosis in untreated patients [10, 11]. Introduction of herceptin therapy has made a remarkable improvement in the survival of these patients. On the other hand, use of other chemotherapeutic and hormonal agents has been a matter of debate. Some authors suggested a decreased sensitivity to anthracycline based chemotherapy in Her2neu amplified breast cancer [12]. Similarly Her2neu over expression also leads to resistance to hormone therapy [13]. Ki67 index and other pathologic parameters may be important factors in these cases in predicting response to therapy. Pathologic parameters and proliferative index has not been widely studied in Her2neu positive breast cancers in our population. Therefore we aimed to compare the prognostic and predictive factors like tumor grade, T-stage, N-stage and ki67 index in Her2neu intrinsic breast cancer subtype with Her2neu over-expressed luminal breast cancers.

## Main text

### Methods

The study included 1951 cases of breast cancers that underwent surgical resection or biopsy at Liaquat National hospital, Karachi Pakistan. The duration of study was 6 years from January 2011 till December 2016. Ethical approval was taken from institutional research and ethical committee. Medical and surgical records of all cases were reviewed. The surgical specimens were grossed according to standard protocols and histopathological examination was done to reveal information regarding tumor size, grade, type and stage. Representative tissue sections were than selected for IHC studies including ER alpha, PR, her2neu and ki67 by DAKO envision method. Following antibodies are used.

1. FLEX Monoclonal Rabbit Anti-human Estrogen receptor alpha, clone EP1, ready to use, purchased from DAKO (code IR084).
2. FLEX Monoclonal Mouse Anti-human Progesterone receptor, clone PgR 636, ready to use, purchased from DAKO (code IR068).
3. Polyclonal Rabbit Anti-human c-erbB-2 oncoprotein, purchased from DAKO (code A0485).
4. FLEX Monoclonal Mouse Anti-human, Ki67 Antigen, Clone MIB-1, Ready to use, purchased from DAKO (code IR626).

One representative tumor block was selected for IHC staining for ER, PR, HER2neu and Ki67. IHC staining was done by DAKO envision method according to manufacturers protocols. Thick sections of 4 mm were deparaffinized in xylene and then dehydrated. Antigen retrieval was done by boiling target DAKO Envision retrieval solution at high ph for 40 min at 96–99 °C. DAKO Envision flex peroxidase blocking reagent was used for blocking endogenous peroxidase activity. The slides were then incubated for 20–30 min in humidity chamber at room temperature followed by incubation with secondary antibody. The substrate (Diamino benzidine + Chromogen) was used producing brown color at the site of target antigen. The hematoxylin was used as a counter stain. Appropriate positive and negative controls were used (normal breast tissue for ER and PR as positive control, known case of her2neu amplified tumor as positive control for her2neu and lymph node as positive control for ki67).

For ER and PR, more than 1% nuclear staining was considered positive expression.

Her2neu IHC scoring was done according to ASCO/CAP guidelines (on a scale of 0 to 3 +). Both intensity of expression and percentage of positively stained cells were taken into account. Only membranous Her2neu expression was evaluated and scored from 0 (negative) to 3+ (positive) according to ASCO and CAP recommendations [14]. Cases with equivocal (2+) IHC expression were further subjected to molecular testing by FISH technique. FISH testing was done utilizing FDA approved Path Vysion Her2 DNA Probe kit purchased from Abbot. Results were given as negative (not amplified) or positive (amplified) according to ASCO/CAP recommendations [14].

Ki67 IHC was scored according to number of positivity stained cells from 0 to 100. Number of positive cells was counted in at least five different areas of the tumor and then average score was calculated.

Her2neu positive luminal and non-luminal breast cancers were defined as follows:

1. Her2neu positive intrinsic (non-luminal) breast cancers: ER and ER negative, Her2neu positive (3+ by IHC or amplified on FISH).
2. Her2neu positive luminal breast cancers: ER positive, Her2neu positive (3+ by IHC or amplified on FISH).

Statistical package for social sciences (SPSS 21) was used for data analysis. Mean and standard deviation were calculated for quantitative variables. Frequency and percentage were determined for qualitative variables. Chi square was applied to see association. *P* value ≤ 0.05 was considered significant.

### Results

**Fig. 1:**
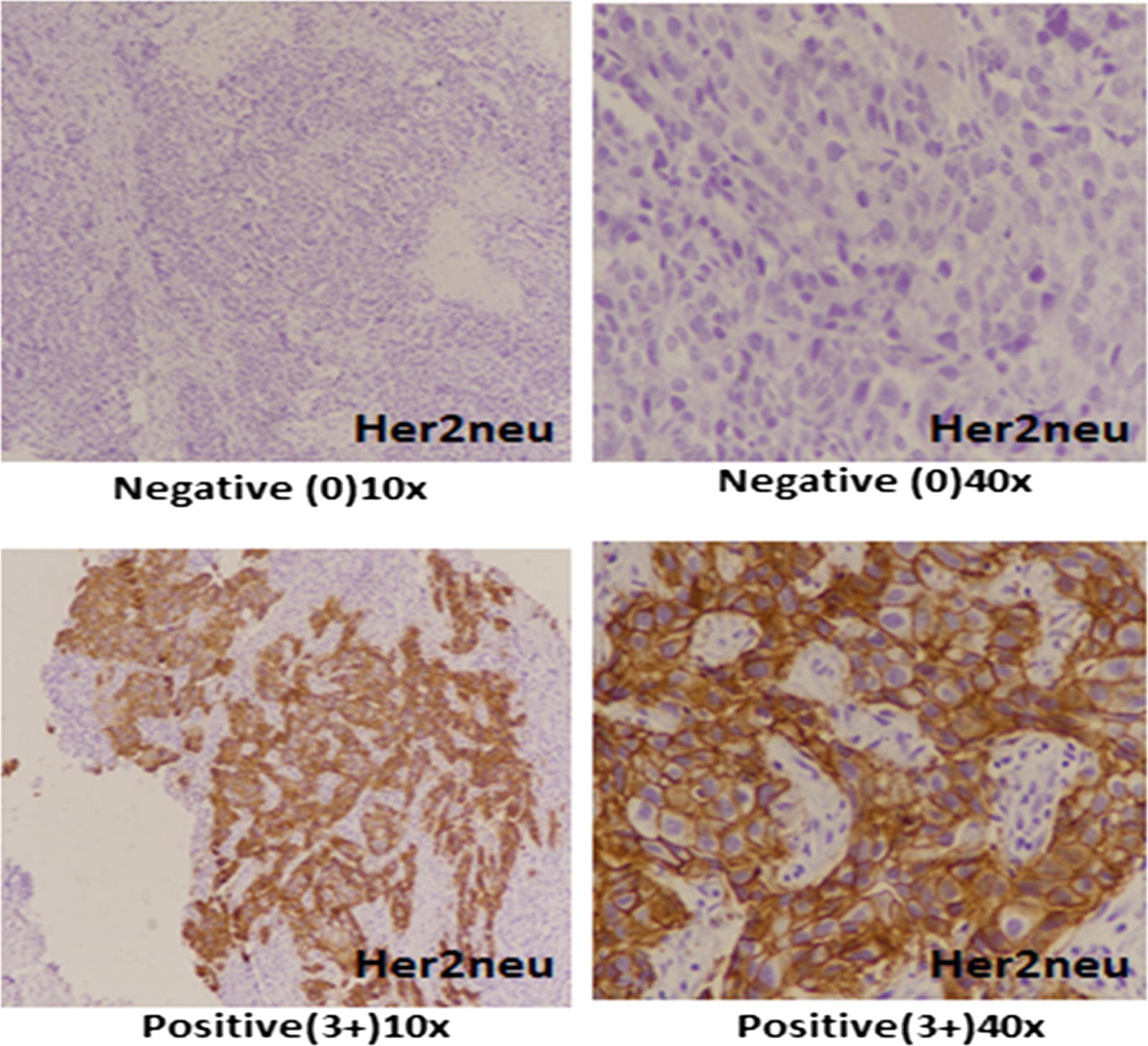
Her2neu expression in breast cancer, positive expression of her2neu (3+) is characterized by strong membranous staining in more than 10% of tumor cells

The study involved 1951 cases of primary breast carcinoma, out of which 801 (41%) cases were Her2neu positive and thus included in the study. Out of these 801 cases, 418 cases (52.2%) showed ER positivity and thus were classified as Her2neu positive luminal cancers whereas 383 cases (47.8%) were ER and PR negative and therefore were labeled as intrinsic Her2neu subtype (non-luminal) (Fig. 1). Table 1 shows association of Her2neu intrinsic subtype (Her2neu+, ER and PR−) with Her2neu positive luminal cancers (Her2neu+, ER+). Significant association was noted in tumor grade and Ki67 index categories where Her2neu positive (non-luminal) cancers showed higher grades and Ki67 proliferative index compared to Her2neu positive luminal cancers. On the other no significant association was noted in T-stage and N-stage. Although mean age of Her2neu intrinsic subtype cancers was low as compared to luminal cancers with Her2neu positivity, no significant association was noted in respect to age groups. Similarly, no significant association was noted when these two categories of Her2neu positive cancers were compared in respect to lymphocytic infiltration of tumor, presence of insitu carcinoma, lymphovascular invasion, dermal lymphatic invasion and pagetoid tumor spread in epidermis (Table 1). Table 2 depicts distribution of histologic subtypes in Her2neu and luminal subtypes of breast cancers. Frequency of lobular and mucinous carcinoma was found to be slightly higher in luminal cancers compared to Her2neu positive non-luminal cancers.

**Table 1:** Association of clinico-pathologic parameters of Her2neu positive intrinsic breast cancer (non-luminal) with Her2neu positive luminal breast cancers (luminal B like)

	n (%)			P-value
	Her2neu positive Intrinsic subtype	Her2neu positive luminal breast cancers	Total	
Age (years)^ab^	49.40 ± 11.649	54.63 ± 12.740	50.98 ± 12.577	< 0.01
Age group (n = 801)				
≤ 30	15 (3.9)	22 (5.3)	37 (4.6)	0.645
31–50	204 (53.3)	216 (51.7)	420 (52.4)	
51–70	148 (38.6)	157 (37.6)	305 (38.1)	
> 70	16 (4.2)	23 (5.5)	39 (4.9)	
Tumor grade (n = 801)				
Grade 1	8 (2.1)	43 (10.3)	51 (6.4)	< 0.01
Grade 2	204 (53.3)	260 (62.2)	464 (57.9)	
Grade 3	171 (44.6)	115 (27.5)	286 (35.7)	
Ki67 mean^ab^	42.61 ± 21.653	21.653 ± 3.344	34.59 ± 23.375	< 0.01
Ki67 index category (n = 801)				
< 15	28 (7.3)	47 (11.2)	75 (9.4)	< 0.01
15–24	64 (16.7)	110 (26.3)	174 (21.7)	
25–44	118 (30.8)	127 (245)	245 (30.6)	
> 44	173 (45.2)	134 (32.1)	307 (38.3)	
Size of tumor^ab^	37.84 ± 16.114	35.21 ± 15.189	36.11 ± 15.233	0.142
Tumor stage (n = 249)				
T1	14 (11.8)	16 (12.3)	30 (12)	0.27
T2	78 (65.5)	95 (73.1)	173 (69.5)	
T3	27 (22.7)	19 (14.6)	46 (18.5)	
Nodal status (n = 250)				
Positive	65 (54.2)	77 (59.2)	142 (56.8)	0.419
Negative	55 (45.8)	53 (40.8)	108 (43.2)	
N stage (n = 250)				
N0	55 (45.8)	54 (41.5)	109 (43.6)	0.172
N1	17 (14.2)	28 (21.5)	45 (18)	
N2	18 (15)	26 (20)	44 (17.6)	
N3	30 (25)	22 (16.9)	52 (20.8)	
Laterality (n = 801)				
Left	193 (50.4)	203 (48.6)	396 (49.4)	0.605
Right	190 (49.6)	215 (51.4)	405 (50.6)	
Lymphocytic infiltration (n = 250)				
Absent	63 (52.5)	81 (62.3)	144 (57.6)	0.218
Moderate	43 (35.8)	40 (30.8)	83 (33.2)	
Severe	14 (11.7)	9 (6.9)	23 (9.2)	
Insitu component (n = 249)				
Present	69 (57.5)	89 (69)	158 (63.5)	0.06
Absent	51 (42.5)	40 (31)	91 (36.5)	
Lymphovascular invasion (n = 250)				
Present	35 (29.2)	47 (36.2)	82 (32.8)	0.24
Absent	85 (70.8)	83 (63.8)	168 (67.2)	
Dermal lymphatic invasion (n = 250)				
Present	15 (12.5)	15 (11.5)	30 (12)	0.815
Absent	105 (87.5)	115 (88.5)	220 (88)	
Pagetoid spread (n = 250)				
Present	7 (5.8)	4 (3.1)	11 (4.4)	0.288
Absent	113 (94.2)	126 (96.9)	239 (95.6)	

Chi Square applied

^a^Mean ± SD

^b^Independent t-Test

**Table 2:** Distribution of histological types of breast carcinoma among Her2neu intrinsic breast cancers (non-luminal) and Her2neu positive luminal breast cancers

	Her2neu intrinsic subtype	Her2neu +ve luminal breast cancers	Total
Ductal (NOS)	363 (94.8)	379 (90.7)	742 (92.6)
Lobular	5 (1.3)	17 (4.1)	22 (2.7)
Cribriform	3 (0.8)	4 (1)	7 (0.9)
Papillary	2 (0.5)	1 (0.2)	3 (0.4)
Mucinous	1 (0.3)	8 (1.9)	9 (1.1)
Micropapillary	0 (0)	3 (0.7)	3 (0.4)
Tubular	4 (1)	0 (0)	4 (0.5)
Metaplastic	5 (1.3)	4 (1)	9 (1.1)
Mixed ductal and lobular	0 (0)	2 (0.5)	2 (0.2)
Total	383	418	801

### Discussion

In the current study we evaluated Her2neu positivity in a large group of patients with breast cancers and impact of ER/PR positivity in these cancers. Frequency of Her2neu positivity was 41% (801/1951 cases) and ER/PR positivity was associated with lower tumor grade and Ki67 index, however there was no significant difference in tumor stage and nodal metastasis.

Four intrinsic molecular subtypes of breast cancer are defined by gene expression analysis studies i.e. luminal A, luminal B, Her2neu enriched and triple negative. St. Gallen international expert consensus on primary therapy of early breast cancer 2013, defined clinicopathological surrogate definitions of these intrinsic breast cancer subtypes including luminal A like, luminal B like, Her2neu positive (non-luminal) and triple negative (ductal) [8]. It is important to recognize two distinct types of luminal B like breast cancers i.e. luminal B like (her2neu negative) and luminal B like (her2neu positive) as her2neu positivity can influence the sensitivity of cancer cells to conventional hormonal and chemotherapy in breast cancer [11, 12].

We found a significantly higher grade and ki67 index in her2neu positive (non-luminal) breast cancers compared to Her2neu positive luminal cancers. This finding is of clinical importance as grade and ki67 index are among predictive factors which can influence sensitivity to chemotherapy. Higher grade and ki67 index have been reported in her2neu positive breast cancers in previous studies [15–19]. On the other hand, we didn’t find any significant difference in T and N stage between Her2neu positive luminal and non-luminal breast cancers, which are considered more important factors determining prognosis in breast compared to grade and ki67 index, which are more of predictive value in breast cancer.

Naeem et al. in a recent study found 12% of breast cancers to be her2neu positive, while all of those were found to have ki67 index more than 14% [20]. Schmidt et al. found that a her2neu score 0 can be considered as a poor prognostic factor in triple negative breast cancer [21]. In another study by Thangarajah et al. it was noted that high ki67 can be used as a poor prognostic factor in terms of disease free survival in hormone receptor positive tumors [22].

Another significant finding in our study is high frequency of her2neu positivity in our cases of breast cancer i.e. 41%; this contrasts to most of the previous studies in other parts of the world where her2neu positivity ranges from 15 to 30% in breast cancer [23, 24]. One of the limitations of our study was that we didn’t evaluate survival and recurrence status in these two subtypes of her2neu positive breast cancers and therefore we suggest that more prospective studies should be done to evaluate if there is any survival benefit of ER/PR positivity in her2neu positive breast cancers.

## Limitations

The main limitation of the study was lack of clinical follow up of patients; however, we found a high frequency of her2neu positivity in our studied population of breast cancer. Moreover, association of her2neu positive (non-luminal) breast cancers with higher grade and ki67 index indicates a predictive value of ER/PR positivity in her2neu positive breast cancers. On the other hand, lack of association with respect to T and N stage, signifies no prognostic benefit of ER/PR in her2neu positive breast cancers.

## Abbreviations

IHC: immunohistochemistry
ER: estrogen receptor
PR: progesterone receptor
